# Construction and Application of an Inducible System for Homogenous Expression Levels in Bulk Cell Lines

**DOI:** 10.1371/journal.pone.0006445

**Published:** 2009-07-30

**Authors:** Jun Yu, Helena Mϋller, Sina Hehn, Steffen Koschmieder, Kai Schönig, Wolfgang E. Berdel, Hubert Serve, Carsten Müller-Tidow

**Affiliations:** 1 Department of Medicine, Hematology and Oncology, University of Münster, Münster, Germany; 2 Preclinical Experiment Center, Fourth Military Medical University, Xi'an, China; 3 Central Institute of Mental Health, Mannheim, Germany; 4 Center of Molecular Biology (ZMBH), University of Heidelberg, Heidelberg, Germany; Victor Chang Cardiac Research Institute, Australia

## Abstract

Stringently controlled conditional expressing systems are crucial for the functional characterization of genes. Currently, screening of multiple clones to identify the tightly controlled ones is necessary but time-consuming. Here, we describe a system fusing Tet (tetracycline)-inducible elements, BAC (bacterial artificial chromosome) and Gateway technology together to allow tight control of gene expression in BAC-transfected eukaryotic bulk cell cultures. Recombinase cloning into the shuttle vector and the BAC facilitates vector construction. An EGFP (enhanced green fluorescent protein) allows FACS (fluorescence activated cell sorting) and the BAC technology ensures tight control of gene expression that is independent of the integrating site. In the current first application, our gene of interest encodes a β-catenin-ERα fusion protein. Tested by luciferase assay and western blotting, in HTB56 lung cancer cells the final BAC E11-IGR-β-catenin-ERα vector demonstrated sensitive inducibility by Tet or Dox (doxycycline) in a dose-dependent manner with low background, and the EGFP was an effective selection marker by FACS in bulk culture HTB56 and myeloblastic 32D cells. This is a highly efficient tool for the rapid generation of stringently controlled Tet-inducible systems in cell lines.

## Introduction

The rapid development of genomic functional research requires stringently controlled expression systems. Tet (tetracycline)-regulated expression systems are widely used. Tet-inducible systems are divided into two classes: one is TetOff, which is constitutively active in the absence of Tet or Dox (doxycycline), whereas off in the presence of Tet or Dox [1], [2]; the other one is TetOn, which becomes active only in the presence of Tet or Dox [3]. One of the advantages of the TetOn system is that, after addition of the inducer, the induction is more rapidly than with the TetOff system following depletion of Tet or Dox. Additionally, gene expression levels can be controlled by adding different doses of inducers [4].

So far, several kinds of vectors have been used to construct Tet or Dox-inducible expression systems such as lentiviral [5], [6], retroviral [7], adeno-associated viral [1], [2], [8]–[11], first-generation adenoviral [2], [9], [10], herpesviral [12], high-capacity adenoviral [13]–[18] and the bacterial artificial chromosome (BAC) vectors [19]. BAC vectors are optimal for harboring relatively long fragments of genomic DNA or large cDNA in excess of 100 kb. They have the considerable advantages of stable propagation and ease of purification. Importantly, as a genomic DNA fragment with effective insulator characteristics, BAC vectors enable the generation of transgenic mammalian cells with predictable expression characteristics independent of the integration site, so they are currently often used in genetic research, such as library construction, transgenic mice production, and gene targeting constructs [20], [21].

However, it is often cumbersome to clone genes into BAC including two steps: first, clone a gene of interest into an intermediate shuttle vector by traditional restriction digestion and ligation; second, the gene of interest is recombined into BAC from the shuttle vector. In the case of multiple expression and functional analysis, proper restriction sites have to be selected according to the different sequences of genes in above-mentioned step I, and then step II is repeated. It is a time- and money-consuming process. However, this work could be simplified by Gateway Technology using lambda phage-based site-specific recombination instead of restriction endonuclease and ligase to insert a gene of interest into the shuttle vector. The DNA recombination sequences (*att*L, *att*R, *att*B, and *att*P) and the LR or BP Clonase enzyme mixtures that mediate the lambda recombination reactions are the foundation of Gateway Technology. In our current study, we cloned a versatile shuttle vector pIGR-RFC. To use this system, the gene of interest is cloned into an entry vector (pENTR), which is then mixed *in vitro* with pIGR-RFC and the Gateway Clonase enzyme mixtures. Site-specific recombination between the *att* sites (*att*R x *att*L) generates an expression clone and a by-product. Thus, the reading frame C (RFC) in the shuttle vector is replaced by the gene of interest [22]. In this way, different genes of interest can now easily be cloned into a universal shuttle vector and then into a BAC.

In our former utilization of Tet-controlled BAC in generating transgenic mice, it appeared that some strains have strong Tet-inducible expression of gene, while others do not, indicating clonal variation of the Tet-controlling system. In order to avoid time- and money-consuming selection of dozens of clones, the enhanced green fluorescent protein (EGFP) may be used as a selection marker because of its convenience for fluorescence activated cell sorting (FACS). By the means of flow cytometry, positive-transfected cells can be easily purified. Thus, in cellular and animal experiments EGFP could act as a useful visible marker by the fluorescence in positive clones.

To the best of our knowledge, our current Tet-controlled expression system is the first combination of Tet-inducible elements, BAC and Gateway technology. It boasts the tight and quantitative expression inducibility by Tet or Dox, the gene cloning convenience by Gateway technology, stable expression in BAC vector and efficient selection for EGFP contributing to a universal technical tool for the analysis of gene expression and its function.

## Methods

### Contruction of pIGR-RFC

**Figure 1:**
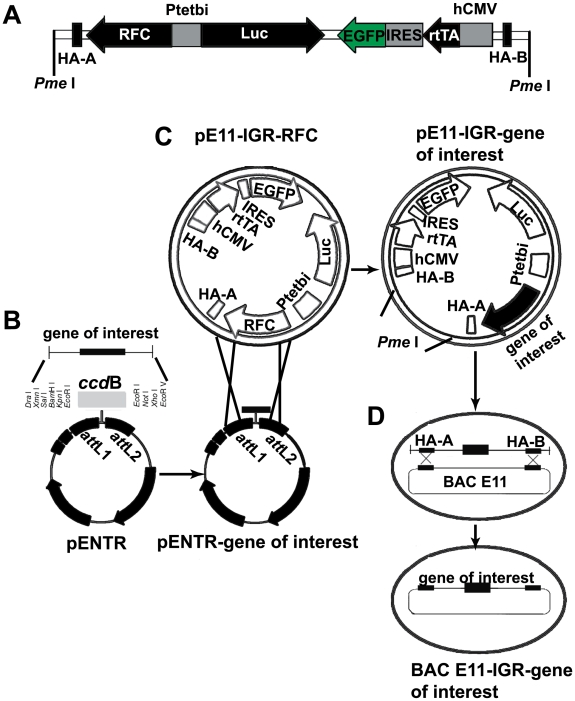
Schematic engineering of the tetracycline-controlled BAC system. (A) Linear depiction of pE11-IGR-RFC vector, which is an intermediate vector including all elements for conditional expression regulated by tetracycline. The promoter P_tet_bi allows simultaneous bidirectional expression: in one direction is the reading frame C (FRC) of Gateway system, into which the gene of interest can be recombined from pENTR plasmid conveniently, and in the other direction is luciferase as a surrogate marker for the expression of the inserted gene of interest. The sequence of rtTA2^S^-M2, a reverse transactivator of P_tet_bi driven by hCMV was also cloned into this shuttle vector. To avoid inserting a third expression cassette, an internal ribosome entry site (IRES) was added between the rtTA2^S^M2 and the EGFP to allow translation and expression of these two proteins from a single promoter hCMV. The homologous arms HA-A and HA-B serve as recombination sites between pE11 and the BAC. (B) A gene of interest is cloned into pENTR vector using proper restriction enzyme sites and the gene of bacteria-toxic protein *ccd*B is replaced. (C) The sites of *att*L1 and *att*L2 in pENTR are recombined with corresponding sites *att*R1 and *att*R2 in RFC respectively. As a result, the gene of interest is engineered into pE11-IGR-RFC and the product is pE11-IGR-gene of interest. (D) The vector pE11-IGR-gene of interest is first linearized by *Pme* I restriction enzyme and then transduced into BAC E11′s host bacteria, within which recombinase is activated by heating (15 min at 42°C). Finally, the gene of interest and all the elements of tetracycline regulation are cloned into BAC.

RFC (Invitrogen, Calsbad, CA, USA), EGFP, IRES and all the elements of Tet-inducible expression system were cloned into pE11.F3.M.F. (established by Dr. K. Schönig, and kindly provided by Dr. H. Bujard), and pIGR-RFC was generated. The map of pIGR-RFC is shown in Figure 1A and C. Its DNA sequence is provided as supplementary material (Supporting Information S1).

### Cell Culture

HTB56 cell line from American Type Culture Collection (ATCC) was cultured in Modified Eagle's medium (Invitrogen, Calsbad, CA, USA) supplemented with 10% Tet-free fetal calf serum (FCS), 1% non-essential amino acids, 1 mM sodium pyruvate, 2 mM l-glutamine, 100 units/ml penicillin and 100 µg/ml streptomycin at 37°C in 5% CO_2_. The 32D cells were cultured and maintained as described earlier [23].

### BAC Transfection

HTB56 cells were seeded 8 hours before transfection into 6-well plates with a density of 300,000 cells per well. Transfection was performed with Lipofectamine transfection reagent (Invitrogen, Carlsbad, CA, USA) by using 1 µg of super-coiled BAC DNA per well. For luciferase assay, 0.1 µg of pRLSV40 encoding renilla luciferase was cotransfected with BAC as internal control to normalize the transfection efficiency. Tet or Dox (Sigma, St. Louis, MO, USA) was added into the culture medium 24 hours after transfection.

Before transfection into 32D cells, the supercoiled BAC DNA was linearized by digestion with I-*Sce*I restriction enzyme (New England Biolabs, Ipswich, MA, USA). For electroporation, 3×10^6^ 32D cells suspended in 300 µl culture medium were transferred into an electrocuvette with a diameter of 4 mm containing 32 µg linearized BAC DNA. The electrical conditions were at 300 V, 6 ms using Pulse Generator EPI 2500 (Fischer, Heidelberg, Germany).

### Dual-Luciferase Activity Assay

Activities of the firefly luciferase and Renilla luciferase in a single sample were measured sequentially using the Dual-Luciferase Report Assay System (Promega, Madison, Wisconsin, USA). Briefly, after 24 hours of Tet or Dox treatment, cells were rinsed twice with phosphate-buffered saline (PBS) and then lysed in 120 µl of passive lysis buffer at room temperature for 15 min. 10 µl of the cell lysate were quickly mixed with 100 µl of Luciferase assay reagent in a luminometer tube. The light emission for the firefly luciferase was recorded for 10 s after a 5 s premeasurement delay using a TD-20/20 luminometer (Turner Designs Instrument, CA, USA). Subsequently, 100 µl of Stop&Glo reagent was added to the same tube to inactivate the firefly luciferase while activating the Renilla luciferase. Variation in transfection efficiency was normalized by dividing the value of the firefly luciferase activity with that of the Renilla luciferase activity.

### Western Blot Analyses

For preparation of whole-cell lysates cells were washed with ice-cold PBS and lysed for 30 minutes on ice in RIPA buffer with 150 mM NaCl as described [24]. Cell lysates were cleared at 20 000 *g* for 10 minutes. After adjustment of protein concentrations, the lysates were boiled in SDS sample loading buffer for 5 minutes and separated by SDS-polyacrylamide gel electrophoresis (PAGE, 4–12%, Invitrogen). Gels were blotted on a polyvinylidene difluoride (PVDF) membrane (Millipore, Bedford, MA, USA) and stained with the anti-human β-catenin first antibody (0.38 µg/ml, BD Biosciences, NJ, USA). Antibody binding was detected with a horseradish peroxidase (HRP)-coupled secondary antibody followed by chemoluminescence detection (ECL Plus, Amersham Pharmacia, Uppsala, Sweden).

### Statistics

Quantitative data are presented as means plus standard deviation (SD). Statistical analyses were performed with SPSS, version 10.0 (SPSS Science, Chicago, IL, USA). Statistical significances of overall differences between multiple groups were analyzed by one-way ANOVA analysis. *P* values of 0.05 or less were considered significant.

## Results and Discussion

To clarify the functional roles of various candidate genes screened by microarray, we established a Tet-inducible expressing system. As shown in Figure 1, the system we are describing here consists of a pENTR vector, an intermediate vector pE11-IGR-RFC and BAC E11. The pE11-IGR-RFC vector accommodates all the elements required for Tet-regulated gene expression including two expression cassettes, one of which encodes the elements responsive to Tet or Dox, and the other one of which encodes the rtTA2^S^-M2, an optimized rtTA construct exhibiting significantly increased sensitivity to Dox [25]. An internal ribosome entry site (IRES) is added between the rtTA2^S^M2 and EGFP to allow expression of these two genes driven by a single hCMV promoter. The EGFP acts as a sorting marker by flow cytometry in eukaryotic cells. The Tet-responsive expression cassette contains a bidirectional promoter P_tet_bi, driving expression of two genes simultaneously. The firefly luciferase is a surrogate marker for the expression of the gene of interest, which can be conveniently recombined into Gateway RFC from a pENTR vector. In the current study, the gene of interest is the β-catenin-ERα fusion gene (Fig. 1B). The backbone of pE11-IGR-gene of interest also comprises two homologous arms HA-A and HA-B sites for recombination with BAC in special bacteria resulting in a destination BAC E11-IGR-gene of interest (Fig. 1D).

**Figure 2:**
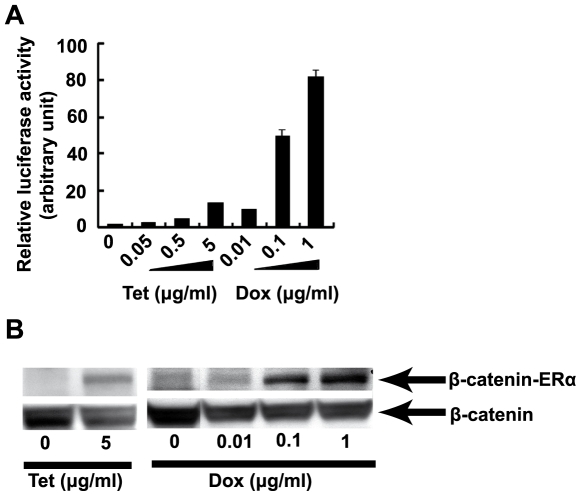
Tetracycline inducibility of BAC E11-IGR-β-catenin-ERα. As a gene of interest, the coding sequence of β-catenin-ERα fusion protein is engineered into BAC. (A) The activity of firefly luciferase, a surrogate marker is assayed to reflect the activity of P_tet_bi. HTB56 cells cultured in 6-well plates (300,000 per well) were cotransfected with 1 µg supercoiled BAC E11-IGR-β-catenin-ERα and 0.1 µg of renilla luciferase-encoding pRLSV40 DNA by lipofectamine reagent. Cells were left untreated or exposed to indicated concentrations of Tet or Dox 24 hours after transfection and luciferase activity was measured in cell extracts 24 hours later. Data from 3 independent experiments are represented as means plus standard deviation (SD). The SD values are too small to be visible in the first five bars. The relative luciferase activity of Tet and Dox untreated cells were set as 1 arbitrarily (* *P*<0.05, ** *P*<0.001 versus cells unexposed to Tet and Dox). (B) The expression of β-catenin-ERα fusion protein induced by Tet or Dox was verified by western blotting with the bands of endogenous β-catenin as loading controls.

Then, tetracycline inducibility of BAC E11-IGR-gene of interest was evaluated. HTEB56 cells transiently transfected by E11-IGR-β-catenin-ERα were exposed to different doses of Tet, Dox or not. We assayed the activity of luciferase, a sensitive surrogate marker reflecting the expression of the gene of interest in the other direction of P_tet_bi. As shown in Figure 2A, there is a dose dependent induction of luciferase activity to 0.05∼5 µg/ml Tet or 0.01∼1 µg/ml Dox. The BAC vector is more sensitive to Dox than to Tet. When the concentration of Dox is increased to 1 µg/ml, the luciferase activity is over 80 times higher than that in the absence of Tet and Dox (*P*<0.001). The dose-dependent inducibility indicated that the expression level of the gene of interest can be regulated deliberately by adding different doses of Dox. In this way, the expression of the gene of interest is controlled not only qualitatively but also quantitatively in transiently transfected bulk cultures.

Furthermore, the inducible expression of β-catenin-ERα was evaluated. In our current study, we used pENTR-β-catenin-ERα as a donor for *in vitro* recombination with Gateway RFC (Fig. 1B). The fusion gene β-catenin-ERα encodes a chimaeric protein comprising wild type β-catenin fused with hormone binding domain of estrogen receptor α. As a result, the DNA fragment of β-catenin-ERα is recombined into BAC. Western blotting was performed after treatment with the indicated concentrations of Dox or Tet for 24 hours. The molecular weight of β-catenin-ERα fusion protein and wild type β-catenin is 136 and 92 kD respectively. Our antibody to β-catenin recognized both β-catenin-ERα fusion protein and the endogenous wild type β-catenin which acted as loading control. In line with the results of the luciferase assay, the expression of β-catenin-ERα was induced by Dox in a dose-dependent manner. Also, Tet (5 µg/ml) induced significant expression of β-catenin-ERα with very low background in non-Tet exposed cells. Importantly, the very low background suggests very little leakiness of inducibility in our current system, which makes it possible to control the expression of the gene of interest in a very stringent mode.

**Figure 3:**
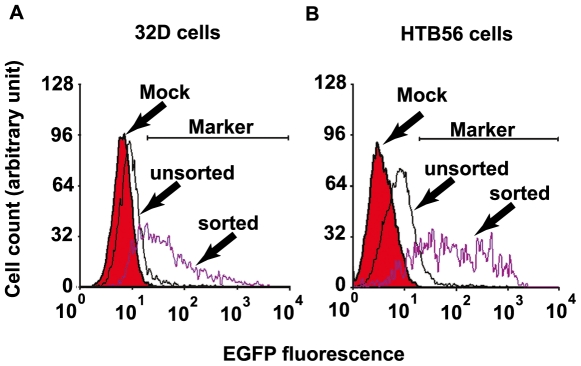
The EGFP is an effective selection marker for FACS. (A) 32D cells were mock transfected or transfected with I-*Sce*I-linearized BAC E11-IGR-β-catenin-ERα DNA by electroporation. The BAC-transfected cells were sorted by FACS 24 hours after transfection and the sorted cells were incubated at 37°C in 5% CO_2_ for another 24 hours. The EGFP fluorescence was compared by flow cytometry. In the range of the indicated marker, there are 0.84, 7.97 and 67.76% of EGFP positive cells in mock, unsorted and sorted cell populations respectively. (B) HTB56 cells were mock transfected or transfected with supercoiled BAC E11-IGR-β-catenin-ERα DNA by lipofectamine reagent. The BAC-transfected cells were sorted by FACS 24 hours after transfection and the sorted cells were incubated at 37°C in 5% CO_2_ for another 24 hours. Compared by flow cytometry, there are 0.16, 9.80 and 79.05% of EGFP positive cells in mock, unsorted and sorted cell populations respectively in the range of the indicated marker.

As a selection marker, EGFP positive cells can be sorted by FACS after transfection. In the final vector BAC E11-IGR-β-catenin-ERα, the expression of EGFP is constitutive and independent of the Tet-regulated cassette. As shown in Figure 3, there are less than 10% green cells 48 hours after transfection, however, around 70% after sorting by flow cytometry. This result indicated that the EGFP is an effective marker for FACS selection. The relatively low transfection efficiency is likely due to the large size (over 100 kb) of the BAC vector. There have been reports of successful transfection of supercoiled BAC DNA into Hela cells with Effectene reagent (Qiagen, Valencia, CA) [26]. In HTB56 cells, we used lipofectamine reagent (Invitrogen). Although the transient transfection efficiency is not high, the low background and high inducibility guarantee usefulness of this BAC system for transient transfection studies. Also, we succeeded in electroporation of linearized (see materials and methods section) BAC E11-IGR-β-catenin-ERα into 32D cells. It is suggested that it is necessary to optimize the transfection conditions in view of cell line specificity. Another cause of the low percentage of green cells is that the final vector BAC E11-β-catenin-ERα is not able to replicate episomally. One of the reasons why the expression of some episomal replicating vectors are not stable over time is that the selection can not distinguish episomal and genomic integrated ones, and the episomal expression is not as stable as that of the integrated [25]. Considering expression stability, we did not design a eukaryotic Ori in the final vector BAC E11-gene of interest. The advantage is that in the stable cell lines we will get, our gene of interest has integrated into the genome compared to the analysis of multiple clones.

In summary, our current Tet-controlled BAC system has tight and dose-dependent inducibility especially to Dox. It is a potential tool to study the functional role of unknown genes in bulk, for example, genes that have been identified in a high-throughput and comprehensive manner.

## Supporting Information

Supporting Information S1 It is the full sequence of the shuttle vector, named pIGR-RFC, in current study.
(0.04 MB DOC)
