# Supplementary material for: Construction and Application of an Inducible System for Homogenous Expression Levels in Bulk Cell Lines
Source: PLoS One. 2009 Jul 30;4(7):e6445. doi: 10.1371/journal.pone.0006445 (PMC2714175; doi:10.1371/journal.pone.0006445)
Supplement: Supporting Information S1 — It is the full sequence of the shuttle vector, named pIGR-RFC, in current study. (0.04 MB DOC) [file pone.0006445.s001.doc]

caattgcatgaagaatctgcttagggttaggcgttttgcgctgcttcgcgatgtacgggccagatatacgcgttgacattgattattgactagttattaatagtaatcaattacggggtcattagttcatagcccatatatggagttccgcgttacataacttacggtaaatggcccgcctggctgaccgcccaacgacccccgcccattgacgtcaataatgacgtatgttcccatagtaacgccaatagggactttccattgacgtcaatgggtggactatttacggtaaactgcccacttggcagtacatcaagtgtatcatatgccaagtacgccccctattgacgtcaatgacggtaaatggcccgcctggcattatgcccagtacatgaccttatgggactttccaaaatgtcgtaacaactccgccccattgacgcaaatgggcggtaggcgtgtacggtgggaggtctatataagcagagctcgtttaaacagaggaacctcaaataggcaagaggcttctaaagaaatgtttgatccggccactttccccggccagaggataggtgtccgcccggcccgggagccctttgcctgagcatcggtgacagacatcttggttccgggactccaccctgagtgttctgcacaggtgagagtgtggaatacaggtaccgggccgaagttcctattcttcaaatagtataggaacttcgggccccccctcgaggtcgacggatccttatcgATGCATCAAGCTTGGTACCGAGCTCGGATCCA CTAGTAACGGCCGCCAGTGTGCTGGAATTCGCCCTT

AAACGGATCGATAGGGAACAAAAGCTGGTGGATCCCCCGGGCTGCAGGAATTCGATATAGTAATCAATTACGGGGTCATTAGTTCATAGCCCATATATGGAGTTCCGCGTTACATAACTTACGGTAAATGGCCCGCCTGGCTGACCGCCCAACGACCCCCGCCCATTGACGTCAATAATGACGTATGTTCCCATAGTAACGCCAATAGGGACTTTCCATTGACGTCAATGGGTGGAGTATTTACGGTAAACTGCCCACTTGGCAGTACATCAAGTGTATCATATGCCAAGTACGCCCCCTATTGACGTCAATGACGGTAAATGGCCCGCCTGGCATTATGCCCAGTACATGACCTTATGGGACTTTCCTACTTGGCAGTACATCTACGTATTAGTCATCGCTATTACCATGGTGATGCGGTTTTGGCAGTACATCAATGGGCGTGGATAGCGGTTTGACTCACGGGGATTTCCAAGTCTCCACCCCATTGACGTCAATGGGAGTTTGTTTTGGCACCAAAATCAACGGGACTTTCCAAAATGTCGTAACAACTCCGCCCCATTGACGCAAATGGGCGGTAGGCGTGTACGGTGGGAGGTCTATATAAGCAGAGCTGGTTTAGTGAACCGTCAGATCCGCTAGCtcgacaattcaccatgtctagactggacaagagcaaagtcataaacggcgctctggaattactcaatggagtcggtatcgaaggcctgacgacaaggaaactcgctcaaaagctgggagttgagcagcctaccctgtactggcacgtgaagaacaagcgggccctgctcgatgccctgccaatcgagatgctggacaggcatcatacccacttctgccccctggaaggcgagtcatggcaagactttctgcggaacaacgccaagtcattccgctgtgctctcctctcacatcgcgacggggctaaagtgcatctcggcacccgcccaacagagaaacagtacgaaaccctggaaaatcagctcgcgttcctgtgtcagcaaggcttctccctggagaacgcactgtacgctctgtccgccgtgggccactttacactgggctgcgtattggaggaacaggagcatcaagtagcaaaagaggaaagagagacacctaccaccgattctatgcccccacttctgagacaagcaattgagctgttcgaccggcagggagccgaacctgccttccttttcggcctggaactaatcatatgtggcctggagaaacagctaaagtgcgaaagcggcgggccggccgacgcccttgacgattttgacttagacatgctcccagccgatgcccttgacgactttgaccttgatatgctgcctgctgacgctcttgacgattttgaccttgacatgctccccgggtaactaagtaaggagagctCAAGCTTCGAATTCTGCAGTCGACGGTACCGCGGGCCCGGCGCCCCTCTCCCTCCCCCCCCCCTAACGTTACTGGCCGAAGCCGCTTGGAATAAGGCCGGTGTGCGTTTGTCTATATGTTATTTTCCACCATATTGCCGTCTTTTGGCAATGTGAGGGCCCGGAAACCTGGCCCTGTCTTCTTGACGAGCATTCCTAGGGGTCTTTCCCCTCTCGCCAAAGGAATGCAAGGTCTGTTGAATGTCGTGAAGGAAGCAGTTCCTCTGGAAGCTTCTTGAAGACAAACAACGTCTGTAGCGACCCTTTGCAGGCAGCGGAACCCCCCACCTGGCGACAGGTGCCTCTGCGGCCAAAAGCCACGTGTATAAGATACACCTGCAAAGGCGGCACAACCCCAGTGCCACGTTGTGAGTTGGATAGTTGTGGAAAGAGTCAAATGGCTCTCCTCAAGCGTATTCAACAAGGGGCTGAAGGATGCCCAGAAGGTACCCCATTGTATGGGATCTGATCTGGGGCCTCGGTGCACATGCTTTACATGTGTTTAGTCGAGGTTAAAAAAACGTCTAGGCCCCCCGAACCACGGGGACGTGGTTTTCCTTTGAAAAACACGATGATAATATGGCCACAACCATGGTGAGCAAGGGCGAGGAGCTGTTCACCGGGGTGGTGCCCATCCTGGTCGAGCTGGACGGCGACGTAAACGGCCACAAGTTCAGCGTGTCCGGCGAGGGCGAGGGCGATGCCACCTACGGCAAGCTGACCCTGAAGTTCATCTGCACCACCGGCAAGCTGCCCGTGCCCTGGCCCACCCTCGTGACCACCCTGACCTACGGCGTGCAGTGCTTCAGCCGCTACCCCGACCACATGAAGCAGCACGACTTCTTCAAGTCCGCCATGCCCGAAGGCTACGTCCAGGAGCGCACCATCTTCTTCAAGGACGACGGCAACTACAAGACCCGCGCCGAGGTGAAGTTCGAGGGCGACACCCTGGTGAACCGCATCGAGCTGAAGGGCATCGACTTCAAGGAGGACGGCAACATCCTGGGGCACAAGCTGGAGTACAACTACAACAGCCACAACGTCTATATCATGGCCGACAAGCAGAAGAACGGCATCAAGGTGAACTTCAAGATCCGCCACAACATCGAGGACGGCAGCGTGCAGCTCGCCGACCACTACCAGCAGAACACCCCCATCGGCGACGGCCCCGTGCTGCTGCCCGACAACCACTACCTGAGCACCCAGTCCGCCCTGAGCAAAGACCCCAACGAGAAGCGCGATCACATGGTCCTGCTGGAGTTCGTGACCGCCGCCGGGATCACTCTCGGCATGGACGAGCTGTACAAGTCCGGACTCAGATCTAGGAGACGACCTTCCATGACCGAGTACAAGCCCACGGTGCGCCTCGCCACCCGCGACGACGTCCCCCGGGCCGTACGCACCCTCGCCGCCGCGTTCGCCGACTACCCCGCCACGCGCCACACCGTCGACCCGGACCGCCACATCGAGCGGGTCACCGAGCTGCAAGAACTCTTCCTCACGCGCGTCGGGCTCGACATCGGCAAGGTGTGGGTCGCGGACGACGGCGCCGCGGTGGCGGTCTGGACCACGCCGGAGAGCGTCGAAGCGGGGGCGGTGTTCGCCGAGATCGGCCCGCGCATGGCCGAGTTGAGCGGTTCCCGGCTGGCCGCGCAGCAACAGATGGAAGGCCTCCTGGCGCCGCACCGGCCCAAGGAGCCCGCGTGGTTCCTGGCCACCGTCGGCGTCTCGCCCGACCACCAGGGCAAGGGTCTGGGCAGCGCCGTCGTGCTCCCCGGAGTGGAGGCGGCCGAGCGCGCCGGGGTGCCCGCCTTCCTGGAGACCTCCGCGCCCCGCAACCTCCCCTTCTACGAGCGGCTCGGCTTCACCGTCACCGCCGACGTCGAGTGCCCGAAGGACCGCGCGACCTGGTGCATGACCCGCAAGCCCGGTGCCCGGGATCCACCGGATCTAGATAACTGATCATAATCAGCCATACCACATTTGTAGAGGTTTTACTTGCTTTAAAAAACCTCCCACACCTCCCCCTGAACCTGAAACATAAAATGAATGCAATTGTTGTTGTTAACTTGTTTATTGCAGCTTATAATGGTTACAAATAAAGCAATAGCATCACAAATTTCACAAATAAAGCATTTTTTTCACTGCATTCTAGTTG ATCGATCCGAAT

AAGGGCGAATTCTG CAGATATCCATCACACTGGCGGCCGCTCGAGCATGCAT

cgattttaccacatttgtagaggttttacttgctttaaaaaacctcccacacctccccctgaacctgaaacataaaatgaatgcaattgttgttgttaacttgtttattgcagcttataatggttacaaataaagcaatagcatcacaaatttcacaaataaagcatttttttcactgcattctagttgtggtttgtccaaactcatcaatgtatcttatcatgtctgctcgaagcggccggccgccccgactctagaattacacggcgatctttccgcccttcttggcctttatgaggatctctctgatttttcttgcgtcgagttttccggtaagacctttcggtacttcgtccacaaacacaactcctccgcgcaactttttcgcggttgttacttgactggcgacgtaatccacgatctctttttccgtcatcgtctttccgtgctccaaaacaacaacggcggcgggaagttcaccggcgtcatcgtcgggaagacctgcgacacctgcgtcgaagatgttggggtgttggagcaagatggattccaattcagcgggagccacctgatagcctttgtacttaatcagagacttcaggcggtcaacgatgaagaagtgttcgtcttcgtcccagtaagctatgtctccagaatgtagccatccatccttgtcaatcaaggcgttggtcgcttccggattgtttacataaccggacataatcataggacctctcacacacagttcgcctctttgattaacgcccagcgttttcccggtatccagatccacaaccttcgcttcaaaaaatggaacaactttaccgaccgcgcccggtttatcatccccctcgggtgtaatcagaatagctgatgtagtctcagtgagcccatatccttgcctgatacctggcagatggaacctcttggcaaccgcttccccgacttccttagagaggggagcgccaccagaagcaatttcgtgtaaattagataaatcgtatttgtcaatcagagtgcttttggcgaagaaggagaatagggttggcaccagcagcgcactttgaatcttgtaatcctgaaggctcctcagaaacagctcttcttcaaatctatacattaagacgactcgaaatccacatatcaaatatccgagtgtagtaaacattccaaaaccgtgatggaatggaacaacacttaaaatcgcagtatccggaatgatttgattgccaaaaataggatctctggcatgcgagaatctcacgcaggcagttctatgaggcagagcgacacctttaggcagaccagtagatccagaggagttcatgatcagtgcaattgtcttgtccctatcgaaggactctggcacaaaatcgtattcattaaaaccgggaggtagatgagatgtgacgaacgtgtacatcgactgaaatccctggtaatccgttttagaatccatgataataattttttggatgattgggagctttttttgcacgttcaaaattttttgcaacccctttttggaaacgaacaccacggtaggctgcgaaatgcccatactgttgagcaattcacgttcattataaatgtcgttcgcgggcgcaactgcaactccgataaataacgcgcccaacaccggcataaagaattgaagagagttttcactgcatacgacgattctgtgatttgtattcagcccatatcgtttcatagcttctgccaaccgaacggacatttcgaagtactcagcgtaagtgatgtccacctcgatatgtgcatctgtaaaagcaattgttccaggaaccagggcgtatctcttcatagccttatgcagttgctctccagcggttccatcttccagcggatagaatggcgccgggcctttctttatgtttttggcgtcttccatggtggctttaccaacagtaccggaatgccaagcttacttagatcgcagatctcgagcccggggctagcacgcgtcagctgactagaggatccccgggtaccgagctcgaattcggggccgcggaggctggatcggtcccggtgtcttctatggaggtcaaaacagcgtggatggcgtctccaggcgatctgacggttcactaaacgagctctgcttatataggcctcccaccgtacacgcctactcgacccgggtaccgagctcgactttcacttttctctatcactgatagggagtggtaaactcgactttcacttttctctatcactgatagggagtggtaaactcgactttcacttttctctatcactgatagggagtggtaaactcgactttcacttttctctatcactgatagggagtggtaaactcgactttcacttttctctatcactgatagggagtggtaaactcgactttcacttttctctatcactgatagggagtggtaaactcgactttcacttttctctatcactgatagggagtggtaaactcgacctatataagcagagctcgtttagtgaaccgtcagatcgcctggagacgccatccacgctgttttgacctccatagaagacaccgggaccgatccagcctccgcggccccgaattcctgcaatgcatttattgtagaagaagaactgcatcaagcttggtaccgagctcggatccactagtaacggccgccagtgtgctggaattcgcccttATCAAACAAGTTTGTACAAAAAAGCTGAACGAGAAACGTAAAATGATATAAATATCAATATATTAAATTAGATTTTGCATAAAAAACAGACTACATAATACTGTAAAACACAACATATCCAGTCCTATGGGCGGCCGCATTAGGCACCCCAGGCTTTACACTTTATGCTTCCGGCTCGTATAATGTGTGGATTTTGAGTTAGGATCCGTCGAGATTTTCAGGAGCTAAGGAAGCTAAAATGGAGAAAAAAATCACTGGATATACCACCGTTGATATATCCCAATGGCATCGTAAAGAACATTTTGAGGCATTTCAGTCAGTTGCTCAATGTACCTATAACCAGACCGTTCAGCTGGATATTACGGCCTTTTTAAAGACCGTAAAGAAAAATAAGCACAAGTTTTATCCGGCCTTTATTCACATTCTTGCCCGCCTGATGAATGCTCATCCGGAATTCCGTATGGCAATGAAAGACGGTGAGCTGGTGATATGGGATAGTGTTCACCCTTGTTACACCGTTTTCCATGAGCAAACTGAAACGTTTTCATCGCTCTGGAGTGAATACCACGACGATTTCCGGCAGTTTCTACACATATATTCGCAAGATGTGGCGTGTTACGGTGAAAACCTGGCCTATTTCCCTAAAGGGTTTATTGAGAATATGTTTTTCGTCTCAGCCAATCCCTGGGTGAGTTTCACCAGTTTTGATTTAAACGTGGCCAATATGGACAACTTCTTCGCCCCCGTTTTCACCATGGGCAAATATTATACGCAAGGCGACAAGGTGCTGATGCCGCTGGCGATTCAGGTTCATCATGCCGTTTGTGATGGCTTCCATGTCGGCAGAATGCTTAATGAATTACAACAGTACTGCGATGAGTGGCAGGCGGGGCGTAATCTAGAGGATCCGGCTTACTAAAAGCCAGATAACAGTATGCGTATTTGCGCGCTGATTTTTGCGGTATAAGAATATATACTGATATGTATACCCGAAGTATGTCAAAAAGAGGTATGCTATGAAGCAGCGTATTACAGTGACAGTTGACAGCGACAGCTATCAGTTGCTCAAGGCATATATGATGTCAATATCTCCGGTCTGGTAAGCACAACCATGCAGAATGAAGCCCGTCGTCTGCGTGCCGAACGCTGGAAAGCGGAAAATCAGGAAGGGATGGCTGAGGTCGCCCGGTTTATTGAAATGAACGGCTCTTTTGCTGACGAGAACAGGGGCTGGTGAAATGCAGTTTAAGGTTTACACCTATAAAAGAGAGAGCCGTTATCGTCTGTTTGTGGATGTACAGAGTGATATTATTGACACGCCCGGGCGACGGATGGTGATCCCCCTGGCCAGTGCACGTCTGCTGTCAGATAAAGTCCCCCGTGAACTTTACCCGGTGGTGCATATCGGGGATGAAAGCTGGCGCATGATGACCACCGATATGGCCAGTGTGCCGGTCTCCGTTATCGGGGAAGAAGTGGCTGATCTCAGCCACCGCGAAAATGACATCAAAAACGCCATTAACCTGATGTTCTGGGGAATATAAATGTCAGGCTCCCTTATACACAGCCAGTCTGCAGGTCGACCATAGTGACTGGATATGTTGTGTTTTACAGTATTATGTAGTCTGTTTTTTATGCAAAATCTAATTTAATATATTGATATTTATATCATTTTACGTTTCTCGTTCAGCTTTCTTGTACAAAGTGGTTCGATaagggcgaattctgcagatatccatcacactggcggccgctcgagcatgcagtgcattgcttcagactccacgggctgctgctggaagacgaaatctgggagccccggtccttcccagctgagatactcttctccccagcccccaataaaagatccagggtacatgattgcgggaaagccggtctccatatgcatgcccgggggatccactagttctagaagctcgctgatcagcctcgactgtgcctctagttgccagccatctgttgtttgcccctcccccgtgccttccttgaccctggaaggtgccactcccactgtcctttcctaataaaatgaggaaattgcatcgcattgtctgagtaggtgtcattctattctggggggtggggtggggcaggacagcaagggggaggattgggaagacaatagcaggcatgctggggatgcggtgggctctatggcttctgaggcggaaagaaccagctggggctcgaggaagttcctattctctagaaagtataggaacttcgcggccgctcgcgcactactcagcgacctccaacacacaagcagggagcagatactggcttaactatgcggcatcagagcagattgtactgagagtcgaccataggggatcgggagatctcccgatccgtcgacgtcaggtggcacttttcggggaaatgtgcgcggaacccctatttgtttatttttctaaatacattcaaatatgtatccgctcatgagacaataaccctgataaatgcttcaataatattgaaaaaggaagagtatgagtattcaacatttccgtgtcgcccttattcccttttttgcggcattttgccttcctgtttttgctcacccagaaacgctggtgaaagtaaaagatgctgaagatcagttgggtgcacgagtgggttacatcgaactggatctcaacagcggtaagatccttgagagttttcgccccgaagaacgttttccaatgatgagcacttttaaagttctgctatgtggcgcggtattatcccgtattgacgccgggcaagagcaactcggtcgccgcatacactattctcagaatgacttggttgagtactcaccagtcacagaaaagcatcttacggatggcatgacagtaagagaattatgcagtgctgccataaccatgagtgataacactgcggccaacttacttctgacaacgatcggaggaccgaaggagctaaccgcttttttgcacaacatgggggatcatgtaactcgccttgatcgttgggaaccggagctgaatgaagccataccaaacgacgagcgtgacaccacgatgcctgtagcaatggcaacaacgttgcgcaaactattaactggcgaactacttactctagcttcccggcaacaattaatagactggatggaggcggataaagttgcaggaccacttctgcgctcggcccttccggctggctggtttattgctgataaatctggagccggtgagcgtgggtctcgcggtatcattgcagcactggggccagatggtaagccctcccgtatcgtagttatctacacgacggggagtcaggcaactatggatgaacgaaatagacagatcgctgagataggtgcctcactgattaagcattggtaagaagttcctattctctagaaagtataggaacttcttaattaacggctagcaaatcacaaataaatgaaagcctgtaaaaagaacagctgatttctcagtgaaaacattcaaagccagaagaaccatagaaatatactccaaatcctaaaagactaagacagccaaactaaggtaacatatccaacaaaactagtgagatgactgaagaagaaggaatatttcccataatataatttccataatataaacagcctctaaaattttgtatgttcaataagtcaaatctaaagaagacacaagaagcattgtttaaacgctagctgcattctagttgtggtttgtccaaactcatcaatgtatcttatcatgtctgtataccgtcgacctctagctagagcttggcgtaatcatggtcatagctgtttcctgtgtgaaattgttatccgctcacaattccacacaacatacgagccggaagcataaagtgtaaagcctggggtgcctaatgagtgagctaactcacattaattgcgttgcgctcactgcccgctttccagtcgggaaacctgtcgtgccagctgcattaatgaatcggccaacgcgcggggagaggcggtttgcgtattgggcgctcttccgcttcctcgctcactgactcgctgcgctcggtcgttcggctgcggcgagcggtatcagctcactcaaaggcggtaatacggttatccacagaatcaggggataacgcaggaaagaacatgtgagcaaaaggccagcaaaaggccaggaaccgtaaaaaggccgcgttgctggcgtttttccataggctccgcccccctgacgagcatcacaaaaatcgacgctcaagtcagaggtggcgaaacccgacaggactataaagataccaggcgtttccccctggaagctccctcgtgcgctctcctgttccgaccctgccgcttaccggatacctgtccgcctttctcccttcgggaagcgtggcgctttctcaatgctcacgctgtaggtatctcagttcggtgtaggtcgttcgctccaagctgggctgtgtgcacgaaccccccgttcagcccgaccgctgcgccttatccggtaactatcgtcttgagtccaacccggtaagacacgacttatcgccactggcagcagccactggtaacaggattagcagagcgaggtatgtaggcggtgctacagagttcttgaagtggtggcctaactacggctacactagaaggacagtatttggtatctgcgctctgctgaagccagttaccttcggaaaaagagttggtagctcttgatccggcaaacaaaccaccgctggtagcggtggtttttttgtttgcaagcagcagattacgcgcagaaaaaaaggatctcaagaagatcctttgatcttttctacggggtctgacgctcagtggaacgaaaactcacgttaagggattttggtcatgagattatcaaaaaggatcttcacctagatccttttaaattaaaaatgaagttttaaatcaatctaaagtatatatgagtaaacttggtctgacag
